# Upper mesophotic depths in the coral reefs of Eilat, Red Sea, offer suitable refuge grounds for coral settlement

**DOI:** 10.1038/s41598-019-38795-1

**Published:** 2019-02-19

**Authors:** Netanel Kramer, Gal Eyal, Raz Tamir, Yossi Loya

**Affiliations:** 1 School of Zoology, Tel-Aviv University, Tel Aviv, 69978 Israel; 2 The Interuniversity Institute for Marine Sciences in Eilat, Eilat, 88103 Israel; 3 Present Address: ARC Centre of Excellence for Coral Reef Studies, The University of Queensland, St. Lucia, QLD 4072 Australia

## Abstract

Due to increasing frequency of disturbances to shallow reefs, it has been suggested that Mesophotic Coral Ecosystems (MCEs, 30–150 m depth) may serve as a refuge for corals and a source of larvae that can facilitate the recovery of shallow degraded reefs. As such, they have received increased attention in the past decade, yet remained understudied regarding recruitment dynamics. Here we describe coral recruitment dynamics on settlement tiles and their adjacent natural habitats (10 m vs. 50 m depths) in Eilat, over a period of 5.5 years. The tiles were deployed along three sites onto 18 racks (3 at each depth and at each site). Recruitment patterns varied both temporally and spatially, ending up to two-fold higher juvenile density and higher recruitment rates at mesophotic sites. Settlement surface preference changed with depth, favoring exposed surfaces in mesophotic waters and cryptic surfaces in shallow waters. Juvenile assemblages differed between depths and were distinct from adjacent natural habitats. Over half of the recruited genera overlapped between depths. We suggest that Eilat MCEs serve as a larval sink, providing settlement grounds for shallow-reef propagules. In view of their significance, we call for the protection of these unique and distinct deep-reef habitats.

## Introduction

Coral reefs are among the world’s most threatened ecosystems. The increasing frequency and severity of disturbances to coral reefs have been leading to a rapid decline in coral cover^1,2^ as recorded worldwide over the past few decades. Consequently, reefs may undergo phase-shifts to less desirable, degraded communities, such as algal-dominated or sponge-dominated states that could exclude or outcompete corals^3,4^. Additionally, the accumulation of stressors results in negative reproductive changes, affecting fecundity and fertilization success^5,6^. These changes ultimately also affect larval dispersal and recruitment, which contribute to the negative cascading effect on coral reefs: i.e., reduction in coral cover; a diminished supply of coral planulae; and, eventually, insufficient recruits for recovery. Recruitment is increasingly interrupted as the intervals between disturbances become shorter and, therefore, recovery time also shortens, inhibiting full recovery^7^. Hence, the persistence, recovery, and resilience of coral reefs largely depend upon crucial early life-history processes: e.g. reproduction, settlement, and recruitment^8,9^.

Most of our knowledge on recruitment is limited to shallow-reef habitats, located between 0–30 m, while the deeper sections of coral reefs have been poorly studied due to diving limitations. These deeper habitats, known as Mesophotic Coral Ecosystems (MCEs)^10^, are typically found at depths ranging from approximately 30–40 m to 150 m. Often highlighted as important ecological refuges, the upper MCE zones (30–60 m) may offer protection from adverse environmental impacts that occur on shallower reefs, an hypothesis referred to as the “deep reef refuge hypothesis” (DRRH)^11^. It has thus been suggested that these deep-reef habitats are connected to the shallow reefs through larval exchange and could function as critical ecological refuges for shallow-reef taxa^11,12^, buffering from major disturbances, such as thermal stress and high light irradiance that cause coral bleaching^13^. The DRRH is currently being extensively tested and has gained both support and reservation^14–19^. The results may be specific to different geographical locations, since there is considerable heterogeneity in the extent to which species overlap between shallow coral reefs and MCEs, and the susceptibility to anthropogenic and natural disturbances varies among the MCEs locations.

Data on the early life-history dynamics, such as recruitment and post-settlement processes of mesophotic corals, are lacking worldwide. Even the well-studied coral reefs of Eilat, at the northern Gulf of Eilat/Aqaba (GoE/A), Red Sea, have limited information available on life history traits^20^. Recovery rates also appear to be largely unknown, seriously hindering effective management of these ecosystems. MCEs may play an important role in settlement choice for coral larvae, as degraded shallow reefs with high algal biomass constitute poor nursery habitats for settling coral recruits^8^, which may search for better nearby habitats more suitable for settlement.

**Figure 1:**
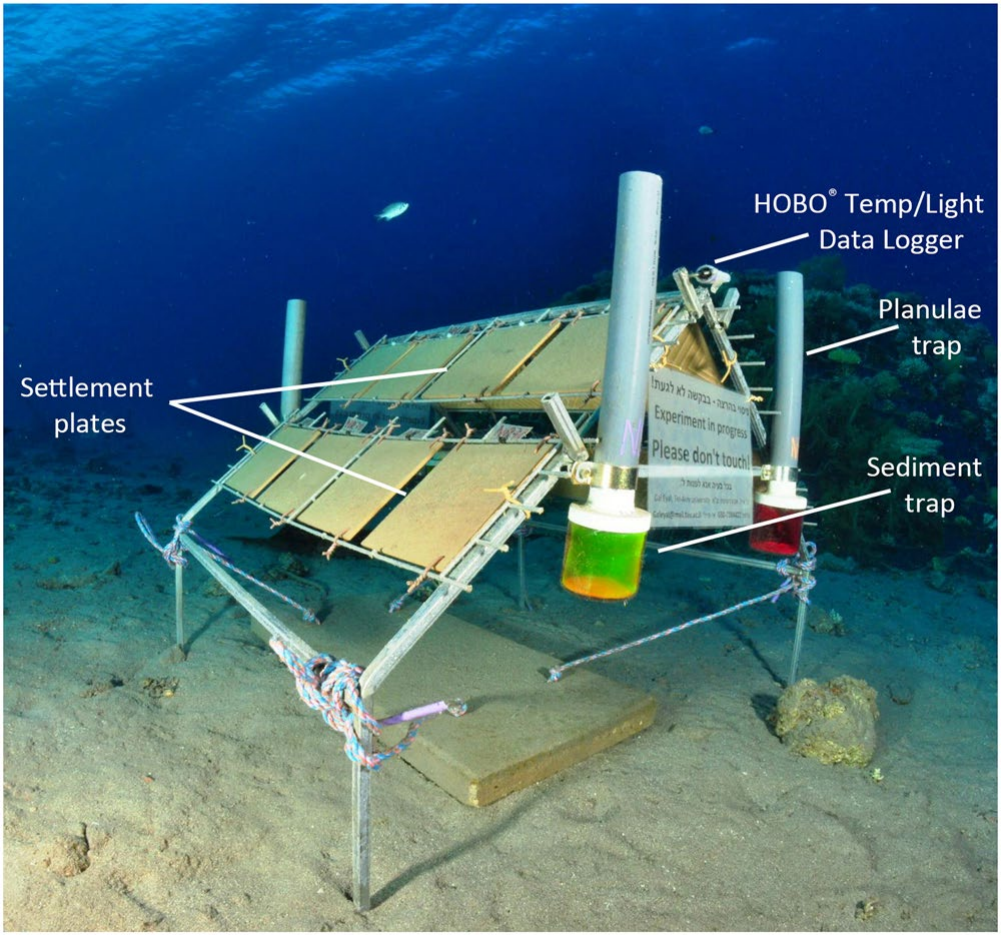
Monitoring rack model featuring 16 terracotta settlement tiles (each 20 × 20 × 1 cm) secured at a 45° angle to the bars; eight facing east (open-sea) and eight facing west (shore). Other components of the rack are not noted in this study.

This study compares the recruitment patterns and survival rates between early life-stage individual corals in MCEs (50 m) and shallow reefs (10 m) in Eilat on settlement racks (Fig. 1). These aspects are currently unknown for mesophotic scleractinian corals. Understanding the primary differences between shallow reefs and MCEs may offer new insights into this ecosystem and its potential role as refugia for depth-generalist species that are under threat in the degraded shallow reefs, as well as provide information for the effective management of marine ecosystems.

Since MCEs feature unique environmental conditions, we sought to test the hypothesis that upper MCEs exhibit different recruitment patterns to those of the shallow reefs. Specifically, we addressed the following questions: (1) Does coral recruitment vary between the shallow reefs and upper MCEs? (2) Are recruit assemblages between the shallow reefs and upper MCEs distinct or similar? (3) Do the recruited genera exhibit a habitat preference in regard to depth? And, (4) how many recruits survive after settlement? Finally, we discuss the potential role of mesophotic recruitment in replenishing shallow reefs and serving as refuge grounds.

## Results

### Recruitment patterns

Over a period of 5.5-yr a total of 1,371 individual corals belonging to 14 families and 27 genera were recorded across the study sites (Table 1). Juvenile coral assemblages at all sites were dominated by the genus *Stylophora* (34% of total recruits), *Porites* (23%) and *Psammocora* (8%). Recruited corals at the mesophotic sites accounted for 62% of all recorded corals in both depths. The majority of mesophotic recruits were represented by *Porites* (35%), and the majority of shallow-reef recruits by *Stylophora* (63%).

**Table 1:** Total abundance of recruited and dead corals at family and genus levels, at shallow (10 m) and mesophotic (50 m) depths.

*Depth Taxa*	Shallow		Mesophotic	
	Recruited	Died	Recruited	Died
**Acroporidae**	**1**		**8**	
*Astreopora*	1		8	
**Agariciidae**	**3**		**21**	
*Leptoseris*	2		8	
*Pavona*	1		6	
**Astrocoeniidae**	**2**		**5**	
*Stylocoeniella*	2		5	
**Coscinaraeida** ***e***	**1**		**19**	
*Coscinaraea*	1		19	
**Dendrophylliidae**	**3**		**15**	**2**
*Rhizopsammia*	3		15	2
**Fungiidae**	**1**		**14**	**7**
*Podabacia*	1		14	7
**Lobophylliidae**	**11**		**9**	
*Acanthastrea*	2		5	
*Echinophyllia*	2			
*Cynarina*			1	
*Leptastrea*	3		3	
*Oxypora*	2			
**Merulinidae**	**67**	**10**	**52**	**4**
*Cyphastrea*	19	2	26	3
*Dipsastraea*	11	5		
*Echinopora*			11	1
*Favites*	26	3	25	1
*Platygyra*	1			
**Milleporidae**	**12**	**3**		
*Millepora*	12	3		
**Pocilloporidae**	**363**	**127**	**145**	**64**
*Pocillopora*	28	5	5	
*Seriatopora*			88	5
*Stylophora*	329	122	132	59
**Poritidae**	**16**	**7**	**297**	**67**
*Porites*	15	7	297	67
*Tubastrea*	1			
**Psammocoridae**	**22**		**82**	**2**
*Psammocora*	22		82	2
**Incertae Sedis**			**9**	**1**
*Blastomussa*			2	
*Plerogyra*			7	1
**Siderastreidae**			**2**	
*Siderastrea*			2	
**Unidentified**	**21**	**18**	**170**	**53**
**Grand total**	**523**	**165**	**848**	**200**

**Figure 2:**
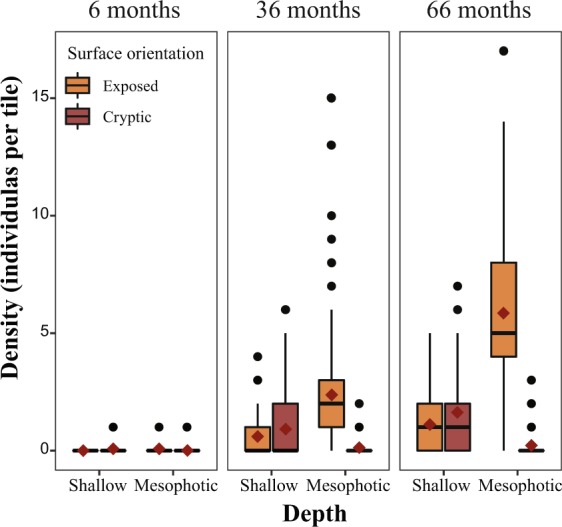
Box plots of juvenile density (juvenile tile^−1^; pooled among all tiles at both depths and all sites; n = 1371) between shallow (10 m) and mesophotic (50 m) after 6, 36, and 66 months. Box plots show the median, interquartile range, ±1.5 interquartile range with dots representing outliers. Red diamonds represent mean values. Significant density difference between depths, surface orientation, and time (permutational ANOVA, *p* < 0.0001).

Density (measured as number of juveniles per tile) varied significantly over time and space (permutational ANOVA, *p* < 0.001; Fig. 2). During the first 24 months of the study, overall juvenile mean density was the same across depths. However, density for the final census was significantly higher on the mesophotic tiles (permutational ANOVA, *p* < 0.001). Mesophotic recruited density reached over two-fold higher than that of the shallow reefs. At the sites DB and OJ (see Methods), density in the mesophotic waters was two-fold and three-fold higher, respectively, compared with the shallow waters. At the NR site density was similar between depths. Mean recruit density was highest at the exposed surfaces of the mesophotic reefs, reaching up to 6 juveniles per tile (5.81 ± 0.31 juveniles per 400 cm^2^; ±SE; Fig. 2). Furthermore, the interaction effect between depth and surface orientation was highly significant (permutational ANOVA, *p* < 0.001) throughout most of the study period, indicating that recruitment on a particular surface orientation was affected by depth. While deep recruits, regardless of the genus, consistently preferred exposed (upper-slope) surfaces, shallow recruits preferred in particular cryptic (under-slope) surfaces. A small portion of the cryptic shallow recruits, mainly Pocilloporidae (22/24), had extended their growth to the exposed surfaces. Tile direction (inshore vs. open-sea) had no effect on recruitment preference.

**Figure 3:**
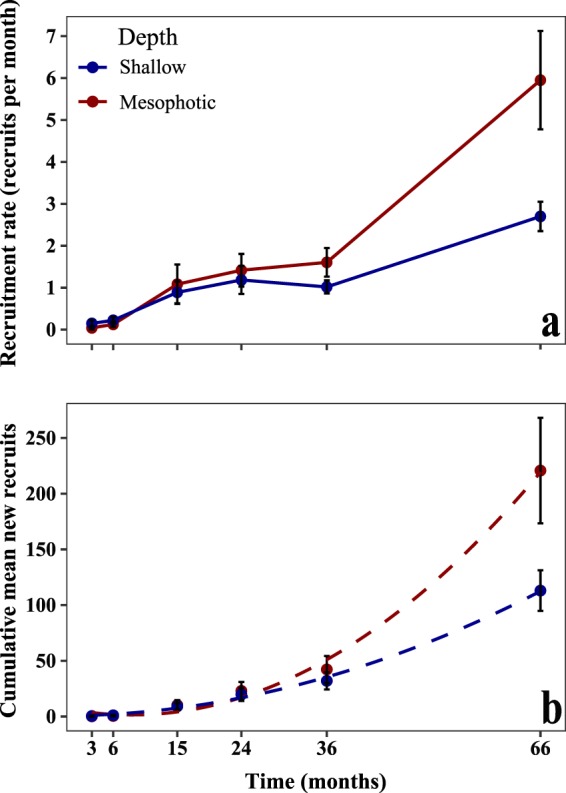
**(a)** Recruitment mean rate (recruits mo^−1^) and (**b**) Cumulative mean of new recruits during the study period per depth (pooled among all rack depths and sites). Dashed trend lines (**b**) illustrate a second order polynomial recruitment at shallow sites (R^2^ = 0.98) and at mesophotic sites (R^2^ = 0.99). Recruitment rates vary between depths and between times (n = 102, permutational ANOVA, *p* < 0.05). Error bars are ±SE.

Mean recruitment rate (measured as the number of new recruits per month) varied between depths and census times (permutational ANOVA, *P* < 0.05; Fig. 3a). Generally, deep racks displayed higher recruitment rates (1.22 ± 0.18 recruits mon^−1^; ±SE) starting at 36 months into the study period, compared with shallow racks (0.76 ± 0.09 recruits mon^−1^; ±SE). The first two years of the study exhibited approximately similar mean recruitment rates at both depths. In the final census, mesophotic recruitment rate was significantly higher than shallow recruitment rate (permutational pairwise test, *P* < 0.01). A similar pattern was seen for the cumulative mean of new recruits (Fig. 3b), with mesophotic recruits displaying faster polynomial growth, resulting in a higher number of cumulative recruits over time. Although growth may seem exponential at first glance, it is likely to be a phase of a polynomial relationship, since we expect density-dependent influences and space availability to reduce the arrival of new recruits with time.

### Community composition

**Figure 4:**
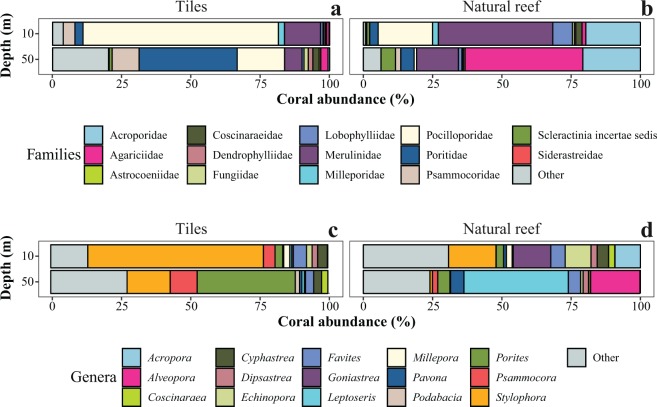
(**a**,**b**) Relative abundance of coral at family level, and (**c**,**d**) relative abundance of the most contributing (70%) genera to the dissimilarity between depths (10 m and 50 m) and communities ((**a**,**c**) tiles, n = 1364; (**b**,**d**) natural reef, n = 4586), as determined by SIMPER analysis.

Overall, 11 families (11/14) consisting of 16 genera (16/27) were identified at both depths, with Merulinidae and Pocilloporidae accounting for most of the shared corals (Table 1). Juvenile abundances were influenced by depth at both family level (Chi-squared test, χ^2^ = 458.4, *p* < 0.001; Fig. 4a) and genus level (Chi-squared test, χ^2^ = 587.2, *p* < 0.001; Fig. 4c). Among the depth-generalist families, over half showed preference for mesophotic waters: Agariciidae, Coscinaraeidae, Dendrophylliidae, Fungiidae, Poritidae and Psammocoridae. For example, 78% of total recruited Psammocoridae were found at mesophotic depths, whereas 73% of total recruited Pocilloporidae settled at shallow depths. Depth-generalist genera exhibiting depth preference include: *Coscinaraea*, *Rhizopsammia*, *Podabacia*, *Porites*, and *Psammocora* settled at mesophotic waters while *Pocillopora* and *Stylophora* settled at shallow waters.

**Figure 5:**
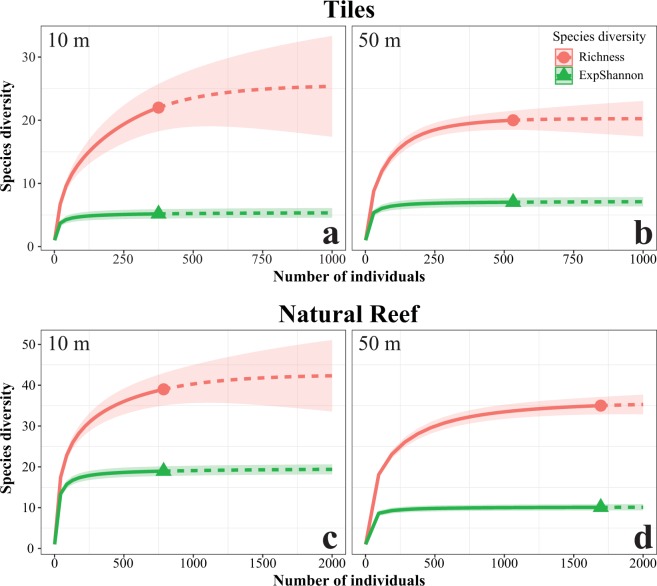
Observed rarefaction and extrapolation curves of two alpha diversity Hill numbers for (**a**,**b**) tiles and (**c**,**d**) natural reef assemblages: richness (q = 0), exponential Shannon (q = 1) diversity metrics, on (**a**,**c**) shallow and (**b**,**d**) mesophotic depths. Solid lines represent the observed accumulation with the number of individuals, and dashed lines represent the extrapolated accumulation. Shaded areas are the 95% confidence intervals. Calculations were performed based on abundance data. No significant differences were observed among the diversities on the tiles between depths (permutational ANOVA, *p* > 0.05). ExpShannon was significantly different in the natural reef between depths (permutational ANOVA, *p* < 0.01).

**Figure 6:**
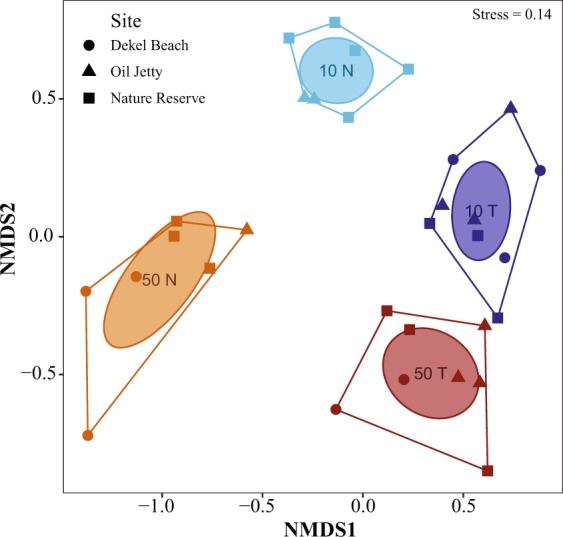
NMDS plot of recruited corals on tiles at 10 m (10 T, *dark-blue polygon*) and at 50 m (50 T, *dark-red polygon*), and of the natural reef community at 10 m (10 N, *light-blue polygon*) and at 50 m (50 N, *orange polygon*). Each dot represents the community composition at a given rack/transect for each of the study sites, based on Bray-Curtis dissimilarity matrices (2D, stress = 0.14). *Ellipses* represent SE for each community.

The first recruits to appear three months after deployments of the tiles were from the genera *Stylophora* in shallow racks and *Plerogyra* in deep racks. In contrast, late recruits (>3 years) comprised *Acanthastrea*, *Goniastrea*, *Pavona*, *Blastomussa*, and *Stylocoeniella* at the deep sites. *Oxypora* and *Stylocoeniella* appeared late among the shallow recruits. Some recruited genera exhibited specific depth preferences for settlement: four coral genera were recorded only at the mesophotic sites: *Blastomussa*, *Cynarina*, *Plerogyra* and *Seriatopora*; While five coral genera were recorded only at the shallow sites: *Dipsastrea*, *Millepora* (reef-building hydrozoan), *Oxypora*, *Platygyra*, and *Tubastrea*. Hill numbers of the two alpha diversity indices were similar between depths for juvenile assemblages on the tiles (permutational ANOVA, *p* > 0.05; Fig. 5a,b). Species richness was between 5–13 for shallow sites and 6–14 for mesophotic sites. However, exponential Shannon (ExpShannon) diversity estimates revealed a much reduced effective diversity. ExpShannon estimates presented 0.6–2.0 and 1.0–2.5 effective species for shallow and MCEs, respectively. Rarefied species richness at shallow sites did not reach saturation, in contrast to the mesophotic sites. Rarefaction and extrapolation values for the natural reef assemblages (Fig. 5c,d) were nearly double those of the recruited corals on the tiles. Shannon diversity reached highest values at 10 m (permutational ANOVA, *p* < 0.01), while species richness was similar between depths (permutational ANOVA, *p* = 0.34). Although we found no statistical difference in alpha diversity at genus level on the tiles between depths, analysis of similarity (ANOSIM) revealed the juvenile community’s compositions at both depths to be significantly distinct (R^2^ = 0.547, *p* < 0.001; Fig. 6). The average similarity percentage between depths was 53.3%, as determined by SIMPER analysis. The contributing genera for 70% of the similarity were *Stylophora* (33.9%), *Porites* (15.94%), *Psammocora* (12.9%), and *Favites* (8.8%). The alpha diversity between rack recruits and natural reef assemblages significantly differed (permutational ANOVA, *p* < 0.001), in addition to highly distinct compositions between both depths (ANOSIM, R^2^ = 0.679, *p* < 0.001), with an average dissimilarity of 74.6%.

### Mortality

**Figure 7:**
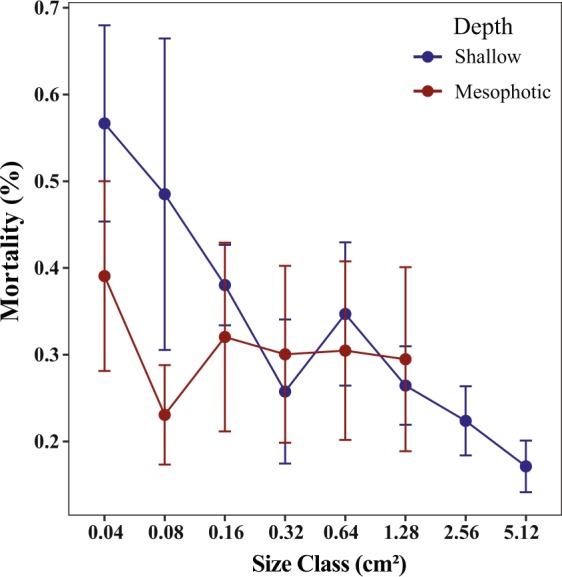
Size-dependent mortality of juvenile corals at shallow (10 m) and mesophotic (50 m) depths (pooled among all rack depths and time). Size classes mortality varied among each other (n = 179, permutational ANOVA, *p* < 0.01) and with the interaction with depth (*p* < 0.05). Error bars are ±SE.

Mortality was moderately low, represented by 27% (365/1371; Table 1) of corals that died or disappeared during the study period. Overall, mortality rates were similar across depths (dead recruit mo^−1^; ±SE; shallow: 0.64 ± 0.21; MCEs: 0.67 ± 0.19). Nonetheless, juvenile mesophotic corals exhibited relatively less mortality (24%) compared with shallow recruits (32%). *Stylophora* experienced 35% mortality at shallow sites and 52% mortality at deep sites, while *Porites* experienced 22% mortality at deep sites, and 47% at shallow sites. There was no significant difference in mean mortality between depths at any site. All other recruited genera presented minor mortalities (≤2%). *Stylophora* experienced 17% higher partial mortality at deep sites (Chi-squared test, χ^2^ = 26.402, *p* < 0.001), and stood out as having the highest partial mortality, compared with the other genera (Table S1). Partial mortality for *Porites* was recorded primarily at the deep sites, with 14% of *Porites* corals undergoing partial mortality. Size-dependent mortality between depths was similar; however, the interaction effect of depth and size class influenced on the mortality of different juvenile size classes (permutational ANOVA, *p* < 0.001; Fig. 7). Shallow sites exhibited significant size-dependent mortality, showing lower mortality with increased size (permutational ANOVA, *p* < 0.01), while mesophotic sites did not show any significance.

## Discussion

The findings from the present study reveal, for the first time, high recruitment success at mesophotic sites, with a more than two-fold higher recruited density than at the shallow reefs (Figs 2 and 3), confirming the hypothesis that recruitment in the upper-MCEs differs from shallow-reef recruitment. Recruitment was clearly shown to vary through time and space, with both depths exhibiting a similar recruitment density for over half of the study period (36 months), but which was eventually higher in the mesophotic reefs (after 66 months). This is also reflected in the total cumulative number of new recruits. The differences found in recruitment between depths could be a result of several possibilities: (1) a higher number of reproductive species and/or reproductive rates within a given depth; (2) differential effects of biotic factors such as predation and competition; and/or abiotic factors such as light, temperature, and flow regimes.

When coral cover is associated with coral recruitment^21,22^, higher coral cover results in a potentially larger supply of larvae due to higher reproduction, and thus contributes to more successful settlement. This of course also depends on the site’s terrain and the availability of suitable substrata for settlement. Shlesinger *et al*.^14^ highlighted the reduced coral reproductive performance with depth in Eilat’s coral reefs, caused by a substantial decrease in fecundity and/or oocyte sizes, which translate directly to lower reproductive rates. In spite of this, mesophotic recruitment was significantly greater. It is possible that the high mesophotic coral cover compensated for the lower reproductive performance, resulting in relatively higher recruitment compared with the shallow sites. This implies that population abundance may be more affected by recruitment dynamics than small changes in fecundity at the individual level. Understanding reproductive rates depends not only on the single colony, but also on viewing the entire range of individuals at a particular depth^23^.

Flow regimes and site proximity may also play a role in mesophotic settlement success. Eilat is located on the western side of the GoE/A and is characterized by down-welling currents created by the Ekman transport, which is caused by the Gulf’s north winds^24^. It is plausible that this higher mesophotic recruitment is achieved partially due to the downwards vertical flow of larvae; i.e., seeding from shallow to mesophotic reefs. Furthermore, the shallow sites at each study location are adjacent to mesophotic sites, separated only by a few dozen meters, and are exposed to the same Gulf waters and other physical conditions (Figs S1 and S2). This is further contributed to the uniquely sharp depth gradient of the Red Sea, in contrast to other MCE sites worldwide that are geographically distinct from shallow reefs^12^. The above combination conditions increase the possibility of larval flow from shallow to mesophotic reefs, and may imply vertical connectivity enabled by downward currents in the GoE/A. Horizontal distance of larval dispersal, dispersing a few hundred meters from place of origin, has been widely investigated^25^, whereas it is only recently that vertical connectivity has begun to receive notice^26,27^. Further research on connectivity is important, as reefs with high levels of connectivity could recover more quickly than isolated reefs^22,28^.

Another factor to consider is that of the high recruitment rates recorded following extreme hot seasons. Recruitment rate burst significantly after 24 months at the mesophotic sites (Fig. 3). A significant increase in the relative number of juvenile colonies has been observed in Eilat’s shallow reefs since 2012^29^, possibly linked to the extreme high temperatures that summer. Increased recruitment of scleractinian corals can be associated with thermal stress^30^, similar to that which occurred following the summer of 2012 in the GoE/A. Elevated temperatures may induce higher reproduction rates in corals, expressed in the release of more coral gametes and larvae into the water column, and shown also to result in polyp bailout^31^. Under such conditions, settlement may occur faster^32^, which consequentially reduces pre-settlement mortality^33^. However, high thermal effects on planulae may be species-specific^34^.

Studies have shown that as depth increases, the abundance of fish decreases, specifically herbivorous fish, apparently due to the reduction in availability of algae in the deep reef ^35^. As a result, coral settlers are less prone to be scraped off the substrate by herbivorous fish grazing on algae. In contrast to the shallow tiles, the mesophotic tiles revealed exceptionally low number of grazing marks, indicating reduced grazing pressure and more available substrate for corals to settle on.

The only exception in regard to recruitment density among the study sites was that of the NR site (the only marine nature reserve in Eilat), which did not differ between depths. The fact that the shallow site is protected and under reservation management may explain this similar pattern between depths. The marine protected area promotes higher shallow coral cover which produces higher reproductive output. Additionally, despite being the only site with lower coral cover at the 50 m depth compared to shallower depths, there was no significant difference in coral recruitment between depths. The site also features the shortest horizontal distance between depths among the study sites (Fig. S2), thus strengthening the likelihood of larval flow between depths. Considering other shallow sites for future reservation management could significantly improve coral recruitment success^36^ and potentially even their recruitment in the MCEs. Similar consideration applies also for mesophotic sites close to densely populated areas, such as in the city of Eilat, since they are particularly vulnerable to negative human impacts^17^.

Coral juveniles do not recruit at random, and depth has a strong effect on the orientation choice of their settlement spots. Either exposed (upper-surfaces) or cryptic (under-surfaces) were favored at each depth, i.e., exposed surfaces in deep waters, and cryptic surfaces in shallow waters^37^ (Fig. 2). In shallow recruitment studies, corals often settle on the cryptic surface^38^. These surfaces are sediment-free and present hidden microhabitats that offer refuge from the effects of algal competition^9,39^ and from indirect predation (mostly by grazers), which can be quickly avoided as the coral recruit size increases^40–42^. The lower densities of recruits found on exposed surfaces affected by sedimentation support previous studies demonstrating the negative effects of sedimentation on recruitment^38,43^. In contrast to shallow recruits, coral recruitment in the MCEs occurred nearly entirely to the upper surfaces of the tiles. Light is probably the factor most related to this shift in orientation^25^. With depth, light may play an important role in settlement orientation, since light intensity is significantly lower at mesophotic depths, and cryptic surfaces are insufficient for growth for most corals, excluding the few corals that can develop in poorly lit environments, such as *Rhizopsammoa* (azooxanthellate) and *Plerogyra* (zooxanthellate). Additionally, this trend to settle on upper surfaces can be attributed to lower predation pressure and competition for space in MCEs, as settling corals do not seek hidden habitats, unlike their shallow recruit counterparts.

Clearly, opportunistic species colonize first (e.g. *Stylophora pistillata*^44^), while slow-growing species with large coralites and massive morphologies settle later, such as members of the Merulinidae (e.g., *Favites*) or Lobophyllidae (e.g., *Acanthastrea*). These latter massive corals are long-lived with low mortality, and can persist for decades in the absence of recruitment^45^, which may explain their relatively low recruitment found in this study (Fig. 4c; Table 1). The presence of existing opportunistic corals may initiate settlement cues to attract low recruiting corals, and the former can be considered as primary founding corals that enhance biodiversity.

More than half of the families were found overlapping between depths, the majority of these families were found to significantly prefer mesophotic depths. Even though these species may not thrive at mesophotic depths, they still raise hope for the survival of some other shallow selected species that are able to live at these depths. In particular, the Pocilloporidae, though preferring shallow depths, was found to be predominant also on the deep tiles. This family provides evidence of larval flow from shallow to mesophotic depths. For example, *Stylophora* had more juveniles in the mesophotic environment (×12) than would be expected based on its abundance in the adult population (Fig. 4c,d). Additionally, *Seriatopora* was not present in the adult communities at 50 m, despite having been found on the settlement tiles at this depth. It is likely that these juveniles’ common origin is from a local larval pool, since these are brooding species, known for recruiting in their local habitat^25^. Hence, our study’s findings may indicate that shallow reefs in the GoE/A may be considered as a local larval supply pool for the upper mesophotic reefs. Some “weedy” Pocilloporids^45^, are described to opportunistically colonize recently disturbed habitats, thus, marking them as top potential candidates from the MCEs for colonizing shallow disturbed areas. Our results present an initial insight into the potential resilience and recovery of these regions if faced with disturbances, as MCEs may exhibit a longer “recruitment window” than shallow reefs^46^. Coral recruitment is a key determinant in future reef success, and the greater a reef’s recruitment success, the greater its potential for future growth and recovery following disturbance.

Early life history studies often aim at understanding the formation and dynamics of adult coral assemblages, with coral-reef demography sometimes being associated with coral recruitment^47^. This was evident in this study. Though depth was important in determining community structure, the coral assemblages at the end of the study period did not coincide with the known adult coral communities in these areas (Figs 4 and 6). The natural shallower assemblages at 10 m possessed a greater Shannon diversity value, yet did not display any difference in species richness with their mesophotic counterparts, is explained by higher evenness at 10 m. At mesophotic depths, the evenness drops as more specialized species dominate the community (e.g., *Leptoseris*, Fig. 4d). Coral communities are dynamic and changing ecosystems, reaching community equilibrium over time^36,48^, which may explain the distinct community compositions and Alpha diversity differences found between the recruited corals and the adult assemblage. To conclude, the recruited coral assemblages on the tiles have not yet reached a community equilibrium, such as found in the adult natural assemblages, and we expect the depth dominant species to take their place as the dominant species on the tiles in the future through competition and intermediate scales of disturbance^49^.

Early coral recruits are small, initially <1 mm in diameter, and are vulnerable to grazing, over-growth, and smothering from sediment for the ensuing 12 months^8^. In this study, by the time that newly-recruited corals were detected, they may already have survived the most vulnerable early life-history stage. Post-settlement mortality rate was considerably low and was found to target mostly *Porites* and *Stylophora*. Mortality is known to vary with size and age of the corals, with younger colonies suffering from greater mortality than older ones under natural conditions^46,50^. Our study has confirmed this pattern of mortality for both shallow and mesophotic juveniles, as mortality was shown to significantly decrease with size (Fig. 7). Altogether, mortality and size-dependent mortality did not differ between depths. This lack of significance for depth may have resulted from low sample sizes for some size classes, especially for mesophotic racks. We believe that further research on mortality rates of coral early-life stages between depths are necessary for more conclusive results. The relatively high partial mortality found for *Porites* and *Stylophora* at the mesophotic depths has several possible reasons (Table S1). Density-dependent recruitment is a likely reason for *Porites*^21^, since it consistently exhibited high coral density per genus, peaking at 15 *Porites* corals per tile. *Stylophora’s* partial mortality is most likely a result of poor competitive capabilities^44^. Furthermore, if *Stylophora* recruits originated from the shallow reefs, they may have experienced partial mortality resulting from an array of the biotic and abiotic conditions they confronted in their new mesophotic environment.

Though deep-reef recruitment comparison studies are sparse, certain recruitment patterns appear consistent among all deep reefs studied to date (>30 m). Nevertheless, there are definite differences between the Indian Ocean reefs, Atlantic reefs, and Red Sea reefs, as well as among reefs within the same region. As in previous studies (Table S2), coral settlement in this study shifted its orientation with depth, from shaded surfaces to exposed surfaces, usually starting at ~25 m. Regarding recruitment abundance, Rogers *et al*.^51^ showed a decrease in recruitment with depth, while Turner *et al*.^37^ showed an increase down to 25 m, and then a dramatic shift to the lowest recruitment at 40 m. Bak and Engel^52^ and Vermeij *et al*.^53^ found the recruitment density along a depth gradient to be similar between the deepest and the shallow sites in Curaçao, Netherlands Antilles, and, similar to our study, they did not find juvenile mortality to differ between depths. These differences suggest that local physical and environmental factors affect recruit distributions, often combined with differences in local species pools. Our study highlights the importance of long-term monitoring of coral recruitment, through high-resolution photography and not retrieving the tiles only after several months. This allows the succession process to take place uninterruptedly, and a study of the changes in recruitment patterns over a long temporal scale and throughout many recruitment cycles.

In conclusion, this study has demonstrated that even if mesophotic corals reveal a lower reproductive performance^14^, MCEs may still thrive and even exhibit higher coral recruitment than those in shallower waters, proving to be a hospitable and fertile habitat for settling corals. We suggest that the upper mesophotic reefs of the GoE/A may serve as a larval sink, and that certain larval taxa could potentially favor deeper waters as a more suitable habitat for settlement, thus offering an optimistic outlook for the survival of these coral taxa. Hence, our results support the DRRH’s first supposition, i.e., that MCEs may constitute refuge grounds for selected shallow-water coral species^16^. However, these results may be specific to certain geographical locations, since not all mesophotic reefs are biologically similar nor exhibit comparable environmental conditions, they do not present equal potential refugia capabilities for selected shallow reef propagules^17^. Therefore, further extensive studies are needed, in different geographical areas, to examine early life-history characteristics of mesophotic corals for filling in some of the gaps in our understanding of these ecosystems. The results of the present work emphasize the need to declare Eilat’s MCEs as marine protected areas. Moreover, in being distinct and unique, mesophotic reefs are in global need of protection in their own right^17^.

## Materials and Methods

### Study Sites

The coral reefs of Eilat cover approximately seven kilometers in length and are largely fragmented. Three reef sites were chosen for this study (Figs S1 and S2), featuring different geomorphologies and disturbance exposures: (1) Dekel Beach (DB) as the most northern MCE in the GoE/A, with an extensive coral reef from 20 m down to 65 m, and grass/sand patches with coral knolls ~20 m distant from each other. DB is exposed to anthropogenic and natural disturbances (e.g., nutrient enrichment and sedimentation). (2) The Oil Jetty (OJ), which is not disturbed by diving activities but is exposed to anthropogenic disturbances (e.g., oil leakage, phosphate enrichment, port contamination & pollution etc.). (3) The Nature Reserve (NR), is the only protected area along the coast, with fringing reefs near the shore and rich coral reefs stretching down to 50 m.

### Monitoring racks

The monitoring racks with the settlement tiles were deployed in July 2011 at the three sites in both the shallow (10 m) and deep (50 m) habitats (Figs 1 and S3). Each monitoring rack was composed of a triangular prism constructed from stainless steel bars with terracotta settlement tiles attached (20 × 20 × 1 cm), eight facing east (open-sea) and eight facing west (shore), oriented at a 45° angle to the bar, and secured together as two units of four tiles, with three cm intervals between tiles (Fig. S3). A total of 18 monitoring racks were constructed for with each site, comprising three racks deployed in the shallow and three in the deep habitats at 10 m intervals. At DB site, one of the deep racks was destroyed by fishermen shortly following deployment.

### Monitoring method

Censuses were carried out during October 2011, February 2012, November 2012, July 2013, July 2014, and the final census in February 2017, using closed-circuit rebreather diving. Photographs were taken with a DSLR camera (Nikon D-90) with underwater housing (Hugyfot) adapted to 100 m depth, high-quality wide-angle lens (Nikkor 12–24 mm f/4.0), shot at a focal length of 24 mm, strobe system (2 × Ikelite Digital Substrobe DS160) and light system (2 × UK Light Cannon Dive Light HID 10 w). Photos were taken on both sides of the tiles for all racks in a consistent order, for easier and accurate follow up during the analyses.

### Ecological surveys

Ecological surveys were conducted using technical diving in the natural reef of the three sites, parallel to the census on the settlement racks. These surveys were used for community composition comparisons with the recruited juveniles on the tiles. Continuous 50 m long belt transects were examined at 10 and 50 m depth, parallel to the shore at each site, between the years 2016–2017. A hundred photo-plots (70 × 50 cm; see camera specifications above) were photographed from each transect. Thirty photo-plots were randomly selected and analyzed to record coral genus abundance, and to obtain coral density data using Photoshop software (Adobe Photoshop CS6, Adobe Inc.).

### Recruitment patterns

In analyzing the photographs, a new recruit was defined as the initial detection of a new juvenile individual on the settlement tile. We termed a coral as juvenile when its surface area did not exceed 20 cm^2^ (determined from a 2.5 cm colony radius, i.e. 2.5^2^ × π). The identification of recruits was at either genus or family level, due to the difficulty in making species-specific identification in the early stages. It can take weeks/months to identify a new recruit, since the early stages of growth are similar among species. Newly detected corals were marked on the images for consistent follow-through. The identified corals were categorized to site, depth, surface orientation, and direction of the tiles on which they were detected. Recruitment rate was calculated by dividing the number of new recruits counted each time by the number of months between surveys. Coral density was calculated as the number of individuals detected on a given tile (400 cm^2^).

### Mortality

Post-settlement mortality was quantified and expressed as the proportion of colonies detected from the images as dead or missing after a given time; and mortality rate was calculated by dividing the number of dead corals by the number of months between surveys. Partial mortality was defined as a coral presenting negative growth. Size-dependent mortality was determined from mortality data, and coral juveniles were distributed over logarithmic size classes, from 0.04 cm^2^ to 5.12 cm^2^ (i.e., size class 0.04 cm^2^ is from 0.02–0.04 cm^2^, size class 0.08 is from 0.04–0.08 cm^2^, etc.).

### Statistical Analyses

To estimate the spatio-temporal variations in recruitment patterns, we modeled density, recruitment rate, mortality rate, and mortality percentage per size (separate tests) for the settlement tiles at all the sites as a function of depth (nested within site), using permutational analysis of variance with a time random effect. Surface orientation (exposed or cryptic) was added as a covariate in the density analyses. The analyses were run using {lme4}^54^ and {predictmeans}^49^ packages in R software^55^. Rarefaction (interpolation) and prediction (extrapolation) of Hill number individual based curves for species richness and exponential Shannon diversity indices were implemented in the R package {iNEXT}^56,57^. Chi-squared tests of goodness of fit and exact binomial tests were used to determine whether the two depths were equally preferred by genera and family levels and for total mortality. Dissimilarity of recruit versus adult assemblages on shallow and mesophotic reefs was calculated using abundance-based Bray-Curtis dissimilarity. The data were evaluated at two taxonomic levels: genus and family. We defined a sample according to the juvenile community observed on each rack (total of 17 racks) and the adult community observed at each transect (total of 14 transects). Sample matrices were log(x + 1) transformed to Bray-Curtis dissimilarity matrices. Pairwise dissimilarities were calculated by ANOSIM, and SIMPER was performed to determine which taxa contributed to the similarity^58^. These tests were run using PRIMER-7 software. Finally, an NMDS plot was made by {vegan} R package^59^, to illustrate the magnitude of dissimilarity in community structure on shallow and mesophotic reefs, between juvenile and adult communities.

## Supplementary information

Supporting Information

## Data Availability

The datasets generated during and/or analyzed during the current study are available from the corresponding author on reasonable request.

## References

[1] Hughes TP (2017). Global warming and recurrent mass bleaching of corals. Nature.

[2] Hughes, T. P. *et al*. Spatial and temporal patterns of mass bleaching of corals in the Anthropocene. *Science* (80-.). **359**, 80–83 (2018). 10.1126/science.aan8048 29302011

[3] Hughes TP (2007). Phase Shifts, Herbivory, and the Resilience of Coral Reefs to Climate Change. Curr. Biol..

[4] Bell JJ, Davy SK, Jones T, Taylor MW, Webster NS (2013). Could some coral reefs become sponge reefs as our climate changes?. Glob. Chang. Biol..

[5] Baird AH, Marshall PA (2002). Mortality, growth and reproduction in scleractinian corals following bleaching on the Great Barrier Reef. Mar. Ecol. Prog. Ser..

[6] Harborne AR, Rogers A, Bozec Y-M, Mumby PJ (2017). Multiple Stressors and the Functioning of Coral Reefs. Ann. Rev. Mar. Sci..

[7] Neal BP (2017). Caribbean massive corals not recovering from repeated thermal stress events during 2005–2013. Ecol. Evol..

[8] Ritson-Williams, R. *et al*. New perspectives on ecological mechanisms affecting coral recruitment on reefs. *Smithson. Contrib. Mar. Sci*. 437–457, 10.5479/si.01960768.38.437 (2009).

[9] Arnold SN, Steneck RS, Mumby PJ (2010). Running the gauntlet: Inhibitory effects of algal turfs on the processes of coral recruitment. Mar. Ecol. Prog. Ser..

[10] Hinderstein LM (2010). Theme section on ‘Mesophotic Coral Ecosystems: Characterization, Ecology, and Management’. Coral Reefs.

[11] Bongaerts P, Ridgway T, Sampayo EM, Hoegh-Guldberg O (2010). Assessing the ‘deep reef refugia’ hypothesis: Focus on Caribbean reefs. Coral Reefs.

[12] Baker, E. K., Puglise, K. A. & Harris, P. T. *Mesophotic Coral Ecosystems - A Lifeboat for Coral Reefs?* (2016).

[13] Muir PR, Marshall PA, Abdulla A, Aguirre JD (2017). Species identity and depth predict bleaching severity in reef-building corals: shall the deep inherit the reef?. Proc. R. Soc. B Biol. Sci..

[14] Shlesinger T, Grinblat M, Rapuano H, Amit T, Loya Y (2018). Can mesophotic reefs replenish shallow reefs? Reduced coral reproductive performance casts a doubt. Ecology.

[15] Semmler, R. F., Hoot, W. C. & Reaka, M. L. Are mesophotic coral ecosystems distinct communities and can they serve as refugia for shallow reefs? *Coral Reefs*, 10.1007/s00338-016-1530-0 (2016).

[16] Bongaerts P, Riginos C, Brunner R, Englebert N, Smith SR (2017). Deep reefs are not universal refuges: reseeding potential varies among coral species. Sci. Adv..

[17] Rocha LA (2018). Mesophotic coral ecosystems are threatened and ecologically distinct from shallow water reefs. Science (80-.)..

[18] Slattery M (2018). The Pulley Ridge deep reef is not a stable refugia through time. Coral Reefs.

[19] Smith TB (2016). Caribbean mesophotic coral ecosystems are unlikelyclimate change refugia. Glob. Chang. Biol..

[20] Eyal, G., Tamir, R., Kramer, N., Eyal-Shaham, L. & Loya, Y. The Red Sea: Israel. in Mesophotic coral ecosystems (eds. Loya, Y., Puglise, K. A. & Bridge, T. C. L.) (Springer, New York (in press), 2019).

[21] Bramanti L, Edmunds PJ (2016). Density-associated recruitment mediates coral population dynamics on a coral reef. Coral Reefs.

[22] Gilmour JP, Smith LD, Heyward AJ, Baird AH, Pratchett MS (2013). Recovery of an Isolated Coral Reef System Following Severe Disturbance. Science (80-.)..

[23] Holstein DM, Smith TB, Gyory J, Paris CB (2015). Fertile fathoms: Deep reproductive refugia for threatened shallow corals. Sci. Rep..

[24] Labiosa RG, Arrigo KR, Genin A, Monismith SG, van Dijken G (2003). The interplay between upwelling and deep convective mixing in determining the seasonal phytoplankton dynamics in the Gulf of Aqaba: Evidence from SeaWiFS and MODIS. Limnol. Oceanogr..

[25] Gleason DF, Hofmann DK (2011). Coral larvae: From gametes to recruits. J. Exp. Mar. Bio. Ecol..

[26] Holstein DM, Paris CB, Vaz AC, Smith TB (2016). Modeling vertical coral connectivity and mesophotic refugia. Coral Reefs.

[27] Serrano, X. M. *et al*. Long distance dispersal and vertical gene flow in the Caribbean brooding coral Porites astreoides. *Sci. Rep*. **6** (2016). 10.1038/srep21619 PMC4761953 26899614

[28] Cowen RK, Paris CB, Srinivasan A (2006). Scaling of connectivity in marine populations. Science (80-.)..

[29] Shaked, Y. & Amatzia, G. *The Israel National Monitoring Program in the Northern Gulf of Aqab*a. (2016).

[30] Edmunds PJ (2017). Unusually high coral recruitment during the 2016 El Niño in Mo’orea, French Polynesia. PLoS One.

[31] Fordyce A, Camp E, Ainsworth D (2017). T. Polyp bailout in Pocillopora damicornis following thermal stress. F1000Research.

[32] Putnam HM, Edmunds PJ, Fan TY (2008). Effect of temperature on the settlement choice and photophysiology of larvae from the reef coral Stylophora pistillata. Biol. Bull..

[33] Ritson-Williams R, Ross C, Paul VJ (2016). Elevated Temperature and Allelopathy Impact Coral Recruitment. PLoS One.

[34] Serrano XM (2018). Effects of thermal stress and nitrate enrichment on the larval performance of two Caribbean reef corals. Coral Reefs.

[35] Brokovich E (2010). Grazing pressure on coral reefs decreases across a wide depth gradient in the Gulf of Aqaba, Red Sea. Mar. Ecol. Prog. Ser..

[36] Mellin C, Aaron Macneil M, Cheal AJ, Emslie MJ, Julian Caley M (2016). Marine protected areas increase resilience among coral reef communities. Ecol. Lett..

[37] Turner, J. A. *et al*. Depth-related patterns in coral recruitment across a shallow to mesophotic gradient. *Coral Reefs*, 10.1007/s00338-018-1696-8 (2018).

[38] Ricardo GF, Jones RJ, Nordborg M, Negri AP (2017). Settlement patterns of the coral Acropora millepora on sediment-laden surfaces. Sci. Total Environ..

[39] Kuffner, I. *et al*. Inhibition of Coral Recruitment by Macroalgae and Cyanobacteria. *Mar. Ecol. Prog. Ser*. **323** (2006).

[40] Davies SW, Matz MV, Vize PD, Kappel C, Brumbaugh D (2013). Ecological Complexity of Coral Recruitment Processes: Effects of Invertebrate Herbivores on Coral Recruitment and Growth Depends Upon Substratum Properties and Coral Species. PLoS One.

[41] Box SJ, Mumby PJ (2007). Effect of macroalgal competition on growth and survival of juvenile Caribbean corals. Mar. Ecol. Prog. Ser..

[42] Christiansen NA, Ward S, Harii S, Tibbetts IR (2009). Grazing by a small fish affects the early stages of a post-settlement stony coral. Coral Reefs.

[43] Birrell CL, McCook LJ, Willis BL (2005). Effects of algal turfs and sediment on coral settlement. Mar. Pollut. Bull..

[44] Loya Y (1976). The Red Sea coral Stylophora pistillata is an r strategist. Nature.

[45] Darling ES, Alvarez-Filip L, Oliver TA, McClanahan TR, Côté IM (2012). Evaluating life-history strategies of reef corals from species traits. Ecol. Lett..

[46] Arnold, S. N. & Steneck, R. S. Settling into an increasingly hostile world: The rapidly closing ‘recruitment window’ for corals. *PLoS One* **6** (2011). 10.1371/journal.pone.0028681 PMC3237487 22194883

[47] Vermeij MJA, Sandin SA (2008). Density-dependent settlement and mortality structure the earliest life phases of a coral population. Ecology.

[48] Connell JH (1978). Diversity in Tropical Rain Forests and Coral Reefs. Science (80-.)..

[49] Luo, D., Ganesh, S. & Koolaard, J. Predictmeans: Calculate Predicted Means for Linear Models. (2014).

[50] Penin L (2010). Early post-settlement mortality and the structure of coral assemblages. Mar. Ecol. Prog. Ser..

[51] Rogers CS, Fitz HC, Gilnack M, Beets J, Hardin J (1984). Scleractinian coral recruitment patterns at Salt River Submariine Caynon, St. Croix, United States Virgin Islands. Coral Reefs.

[52] Bak, R. P. M. & Engel, M. S. Distribution, abundance and survival of juvenile hermatypic corals (Scleractinia) and the importance of life history strategies in the parent coral community. *Mar. Biol*., 10.1007/BF00395440 (1979).

[53] Vermeij, M. J. A., Bakker, J., van der Hal, N. & Bak, R. P. M. Juvenile coral abundance has decreased by more than 50% in only three decades on a small Caribbean Island. *Diversity*, 10.3390/d3030296 (2011).

[54] Bates D, Mächler M, Bolker B, Walker S (2015). Fitting Linear Mixed-Effects Models Using {lme4}. J. Stat. Softw..

[55] Team, R. C. R: A Language and Environment for Statistical Computing. R Foundation for Statistical Computing, Vienna, Austria. https://www.R-project.org/ (2017).

[56] Hsieh, T. C., Ma, K. H. & Chao, A. iNEXT: an R package for rarefaction and extrapolation of species diversity (Hill numbers). *Methods Ecol. Evol*., 10.1111/2041-210X.12613 (2016).

[57] Chao A (2014). Rarefaction and extrapolation with Hill numbers: A framework for sampling and estimation in species diversity studies. Ecol. Monogr..

[58] Clarke KR (1993). Non‐parametric multivariate analyses of changes in community structure. Aust. J. Ecol..

[59] Oksanen, J. *et al*. Vegan: Community Ecology Package. R package version 2.4-6 (2018).

