# Supplementary material for: Upper mesophotic depths in the coral reefs of Eilat, Red Sea, offer suitable refuge grounds for coral settlement
Source: Sci Rep. 2019 Feb 19;9:2263. doi: 10.1038/s41598-019-38795-1 (PMC6381148; doi:10.1038/s41598-019-38795-1)
Supplement: Supplementary file 1 — Supporting Information [file 41598_2019_38795_MOESM1_ESM.pdf]

Supplementary for

Upper Mesophotic depths in the coral reefs of Eilat, Red Sea, offer suitable refuge grounds for coral settlement

Netanel Kramer[*], Gal Eyal, Raz Tamir, Yossi Loya

[*] Corresponding author: Nati Kramer, email: nati.kramrer@gmail.com

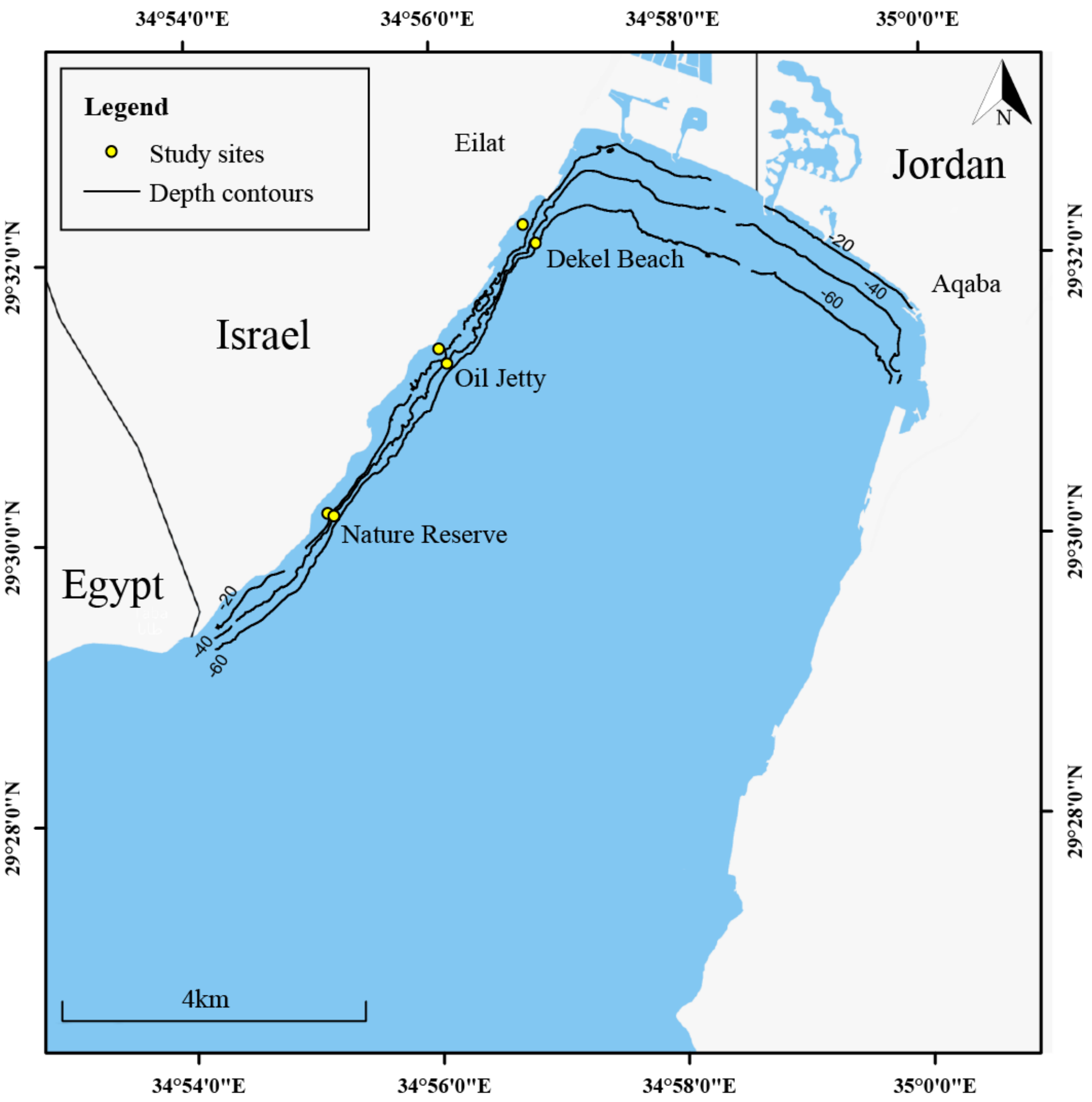

Figure S1. Map of the Gulf of Eilat/Aqaba and the locations of the three study reefs.

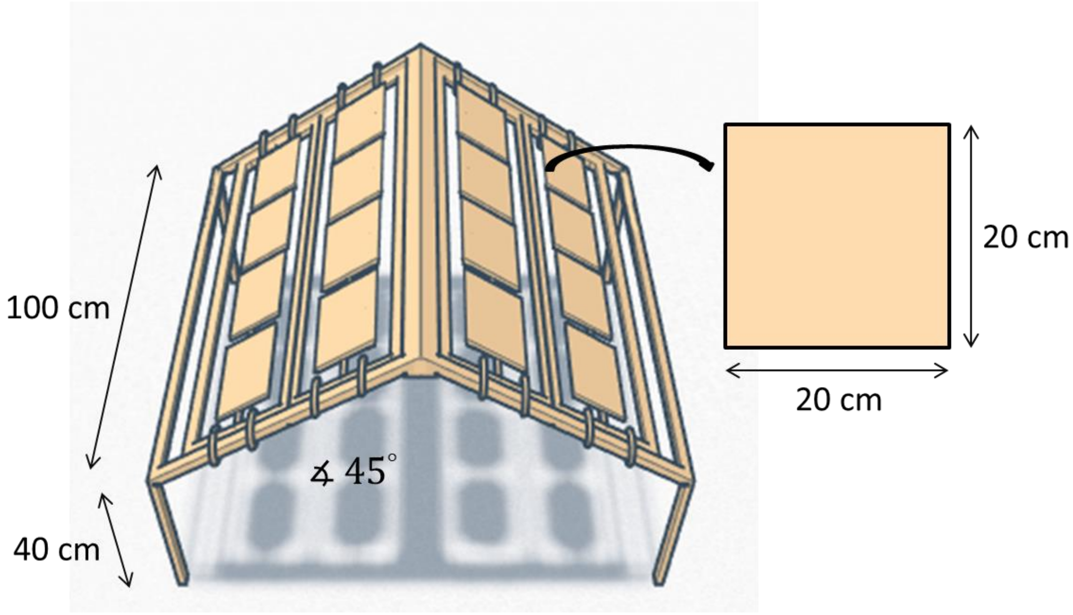

Figure S2. Dimension of the settlement rack model

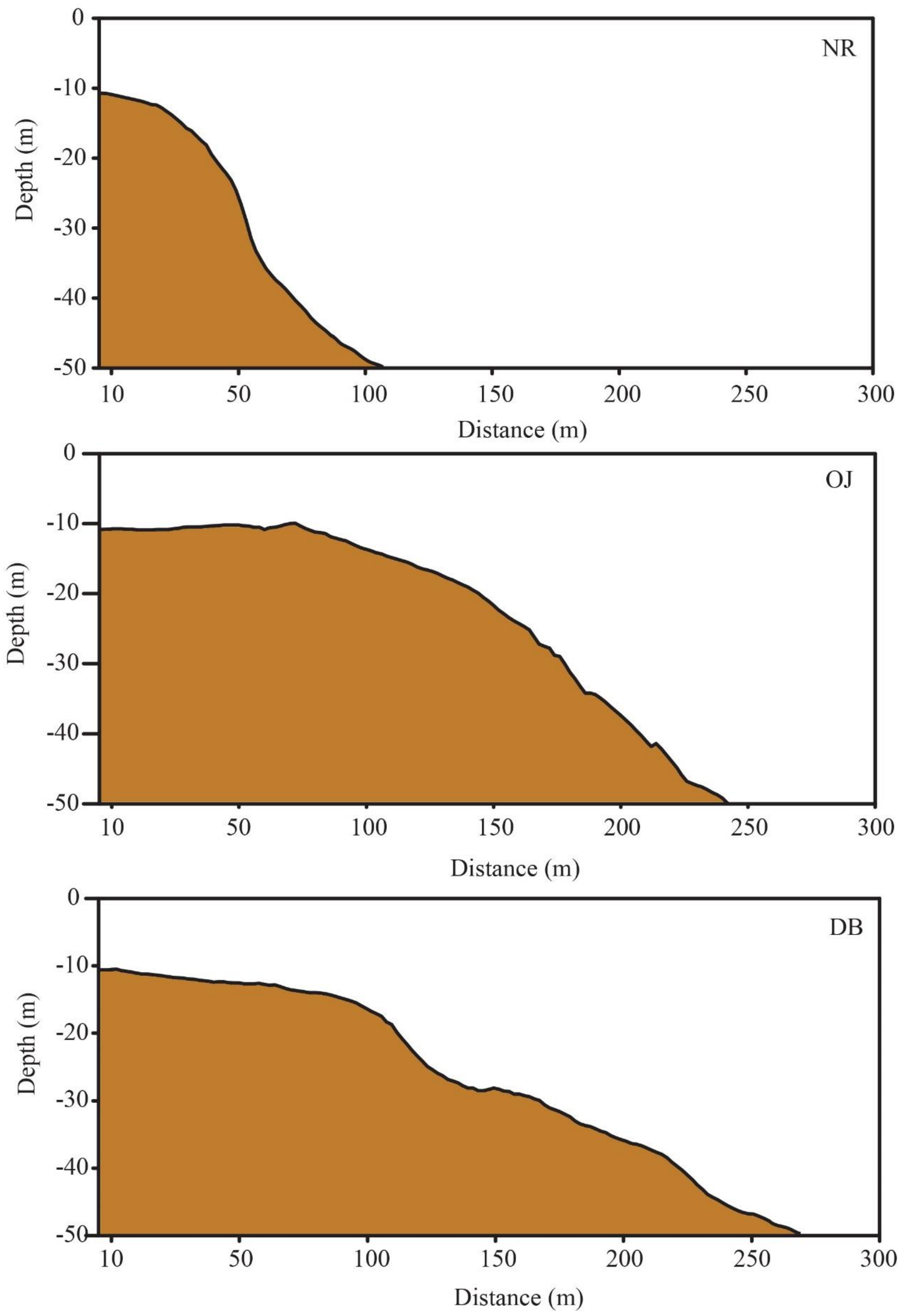

Figure S3. – Relation between depth and distance from shore for each of the three study sites.
Incline angles are: NR – 27.3^{o}; OJ – 11. 5°; DB - 10.3^{o}

Table S1. No. of stony taxa (scleractinian and non-scleractinian) experiencing partial mortality at family and genus levels, at shallow (10 m) and mesophotic (50 m) depths.

Taxa	**Depth**	
	Shallow	Mesophotic
**Acroporidae**		
Astreopora		
**Agariciidae**		**1**
Leptoseris		
Pavona		1
**Astrocoeniidae**		
Stylocoeniella		
**Coscinaraeidae**	**1**	**2**
Coscinaraea	1	2
**Dendrophylliidae**	**1**	
Rhizopsammia	1	
**Fungiidae**		
Podabacia		
**Lobophylliidae**		
Acanthastrea		
Echinophyllia		
Cynarina		
Leptastrea		
Oxypora		
**Merulinidae**		**2**
Cyphastrea		1
Dipsastraea		
Echinopora		
Favites		1
Platygyra		
**Milleporidae**		

Millepora		
**Pocilloporidae**	**22**	**33**
Pocillopora		
Seriatopora		1
Stylophora	22	32
**Poritidae**	**1**	**43**
Porites	1	43
Tubastrea		
**Psammocoridae**		**2**
Psammocora		2
**Incertae Sedis**		**5**
Blastomussa		
Plerogyra		5
**Siderastreidae**		
Siderastrea		
**Unidentified**		**7**
Grand total		

Table S2. Comparison summary of methods used in shallow to mesophotic depth recruitment research.

Location	Depths (m)	No. of Sites	Study duration (months)	Tiles/transects per depth; Dimensions	Definition	Method	Coral identification	Reference
Curaçao, Netherlands Antilles	3-9, 9-17, 17-26, 26-37	2	12	1<br>15 m	≤ 40mm	1 m^2 quadrats along 15 m transect	*In –situ* observation	Back and Engel (1979)[53]
Salt River submarine canyon, St. Croix, USVI	9, 18, 27, 37	2	26	48 tiles<br>120 cm^2	Newly settled	Slabs of *Acropora palmata* bolted to plastic base secured to the substrate	Tile retrieval	Rogers C. S. et al (1984)[52]
Curaçao, Netherlands Antilles	3-9, 9-17, 17-26, 26-37	6	12	≥ 8 transects<br>5 m	≤ 40mm	0.25 m^2 quadrats along a 5 m long transect	*In –situ* observation	Vermeij et al. (2011)[54]
Ningaloo marine park, Western Australia	3 ,8 ,25, 40	3	4	20 tiles<br>11 x 11 x 1 cm	Newly settled	Direct-attachment method (Fig. 1a). Terracotta tiles	Tile retrieval	Turner et al. (2018)[37]
GoE/A, Israel	10, 50	3	66	48 tiles<br>20 x 20 x 1 cm	Newly settled	Triangular prism constructed from stainless steel bars (Fig. 1). Terracotta tiles	High resolution photography	This study
